# Gene editing the phytoene desaturase alleles of Cavendish banana using CRISPR/Cas9

**DOI:** 10.1007/s11248-018-0083-0

**Published:** 2018-07-09

**Authors:** Fatima Naim, Benjamin Dugdale, Jennifer Kleidon, Anthony Brinin, Kylie Shand, Peter Waterhouse, James Dale

**Affiliations:** Centre for Tropical Crops and Biocommodities, Queensland University of Technology, Brisbane, QLD 4000 Australia

**Keywords:** Genome editing, PDS, Cavendish banana, CRISPR/Cas9

## Abstract

Bananas are a staple food source and a major export commodity worldwide. The Cavendish dessert banana is a triploid AAA genome type and accounts for around 47% of global production. Being essentially sterile, genetic modification is perhaps the only pathway available to improve this cultivar. In this study, we used the CRISPR/Cas9 gene editing system to deliver a self-cleaving polycistronic guide RNA (gRNA) designed to target exon 1 of the *Phytoene desaturase* (*PDS*) gene in the Cavendish cultivar “Williams”. Genotyping of 19 independent events showed a 100% *PDS* modification rate primarily in the form of insertions (1–105 nt) or deletions (1–55 nt) (indels) at the predicted cleavage site. Tri-allelic disruptive modifications were observed in 63% of plants and resulted in both albinism and dwarfing. Pale green (16%) and wildtype green (21%) phenotypes generally correlated with in-frame indels in at least one of the three *PDS* alleles. Editing efficiency was dependent on both target site selection and Cas9 abundance. This is the first report of a highly effective CRISPR/Cas9 modification system using a polycistronic gRNA in Cavendish banana. Such an editing platform will be of considerable utility for the development of disease resistance and novel agro-traits in this commercially important cultivar into the future.

## Introduction

Bananas are a perennial, herbaceous monocotyledonous plant grown commercially and by subsistence farmers in the wet tropics and sub-tropics as both a food and fibre crop. Their fruit can be eaten raw or cooked and serve as a staple starch and food source for millions throughout the world. Nearly half of the bananas grown commercially belong to the Cavendish group, a triploid “AAA” genome type. In 2016, global banana exports were valued at USD 8 billion and it is ranked the most popular fruit in industrialised countries (FAO 2017 (http://www.fao.org/economic/est/estcommodities/bananas/bananafacts/en/#.Wz18QRSbFjY); Lescot 2011). Cavendish bananas are parthenocarpic, resulting in a seedless fruit, and are clonally propagated from suckers or in tissue culture. The Cavendish grown today is practically a genetic replica of its progenitor discovered and first propagated over 180 years ago and the varieties cultivated *en masse*, such as “Grand Nain”, are likely natural clonal variants selected for their desirable agronomic traits (D’Hont et al. 2012a). Being essentially sterile, breeding for the introduction of new and useful traits into Cavendish remains virtually impossible.

Genetic modification is perhaps the only avenue available to improve the Cavendish banana, and considerable progress has been made in the development of effective *Agrobacterium*-mediated transformation and plant regeneration systems for this cultivar (Khanna et al. 2004). Recent advances have seen the introduction of novel agro-traits into banana including elevated micronutrient content (Paul et al. 2017), delayed fruit ripening (Elitzur et al. 2016) and engineered resistance to pathogens including viruses (Shekhawat et al. 2012), fungi (Dale et al. 2017), and bacteria (Tripathi et al. 2014). In addition, extensive banana bioinformatics resources are now available in the form of the Banana Genome Hub (http://banana-genome-hub.southgreen.fr/), which provides a valuable bridge for comparative genome analysis, metabolic pathway profiling and the identification of potentially valuable genes and their expression patterns (D’Hont et al. 2012b).

Genome editing technologies are valuable for exploring the underlying mechanisms of gene function and regulation and can serve as a platform for the genetic improvement of crops by removing undesirable chromosomal DNA, up- or down-regulating endogenous genes and introducing novel coding sequences. CRISPR/Cas9 genome editing has proven to be precise, highly effective and versatile in a number of dicotyledonous and monocotyledonous species (reviewed in Bortesi and Fischer 2015). Of the three CRISPR/Cas9 systems (I, II, and III), the type II system from *Streptococcus pyogenes* has been most utilised and comprises two main components, the Cas9 endonuclease and a single guide RNA (gRNA), which includes a 20 nucleotide spacer sequence targeting the gene of interest and a non-variable Cas9 binding site (Mali et al. 2013).

Phytoene desaturase (PDS) is a key enzyme in the carotenogenic pathway and its disruption causes albinism and dwarfing by impairing chlorophyll, carotenoid, and gibberellin biosynthesis. As such, the *PDS* gene has been used as a convenient indicator for CRISPR/Cas9-mediated gene knockouts in a variety of plant species (Fan et al. 2015; Nishitani et al. 2016; Pan et al. 2016). Here, we investigated a CRISPR/Cas9 multiplex gene editing strategy to modify the *PDS* gene of Cavendish banana. We employed the glycine tRNA processing system in order to deliver a polycistronic gRNA (Xie et al. 2015) comprising tandem *PDS* target sequences into banana cells. This approach resulted in a 100% chromosomal *PDS* modification rate and sequencing confirmed triallelic deletions or insertions, which generally correlated with the strength of the albino phenotype.

## Materials and methods

### Isolation of the Cavendish PDS genomic DNA sequence

Total genomic DNA was isolated from banana (Cavendish cv. Williams) leaf material using the CTAB method (Stewart and Via 1993). Primers were designed based on the *Musa acuminata* AAA Group cultivar Cavendish phytoene desaturase (PDS) mRNA sequence (GenBank Accession JQ762260). Genomic DNA (1 µg) was used as a template for PCR with the GoTaq^®^ Green PCR Master Mix system (Promega) and the primers PDS-F2 (5′-TTTAATTATCGATTGTAGATAGACC-3′) and PDS-R2 (5′-GAGGGCTGGCACCATGTCTTGGCT-3′). PCR cycle conditions were as follows: an initial denaturation step at 95 °C for 5 min, followed by 25 cycles of 95 °C for 30 s, 50 °C for 30 s, and 72 °C for 30 s, followed by a final extension at 72 °C for 7 min. PCR products were ligated into pGEM^®^-T Easy (Promega) as per the manufacturer’s specifications. Clones were sequenced using the BigDye Terminator v3.1 sequencing system (Thermo Fisher) at the Queensland University of Technology, Central Analytical Research Facility (QUT CARF). Sequence was analysed using Vector NTI Advance 11 software (Thermo Fisher Scientific).

### Construction of CRISPR/Cas9 vectors

Two CRISPR/Cas9 vectors were assembled targeting the Cavendish PDS gene. The first, construct contained the maize polyubiquitin 1 (Ubi1) promoter directing expression of the human codon optimised *SpCas9* gene and nopaline synthase (nos) termination sequence. The second construct, contained the CaMV 35S promoter directing expression of the *SpCas9* gene and nos terminator. Both cassettes were assembled in pCAMBIA1300 using a Gibson cloning strategy (New England Biolabs, NEB) according to the manufacturer’s specifications. The rice U3 small nucleolar RNA promoter was selected to direct expression of the gRNAs. Two gRNAs targeting Cavendish *PDS* were inserted downstream of the U3 promoter using the tRNA-gRNA system and BsaI cloning strategy described by Xie et al. (2015). For gRNA1, primers S1 (5′-TAGGTCTCCCATGAAGTTAAGGTTTTAGAGCTAGAAATAG-3′) and AS1 (5′-TAGGTCTCACATGGGAGAGACTGCACCAGCCGGGAATC-3′) were used and for gRNA2, primers S2 (5′-TAGGTCTCCGAAGCCAAGACAGTTTTAGAGCTAGAAATAG-3′) and AS2 (5′- ATGGTCTCACTTCTTGTCCTTTGCACCAGCCGGGAATC-3′) were used. The resulting constructs were named pUbi-Cas9-PDS and p35S-Cas9-PDS, respectively. Vectors were mobilised into *Agrobacterium tumefaciens* strain AGL1 by electroporation for banana transformation.

### Banana transformation and plant regeneration

*Musa acuminata* Cavendish cv. Williams (AAA subgroup) embryogenic cell suspensions were prepared from immature male flowers and *Agrobacterium*-mediated transformation performed as described by Khanna et al. (2004) with minor modifications; no centrifugation step was used during Agrobacteria co-cultivation, banana cells were maintained on BSM media for only 1 month (instead of three) post-transformation, and hygromycin (Sigma-Aldrich) antibiotic (5–10 mg/L) was used to select transformed cells. Proliferating embryos were separated in order to ensure each regenerated plantlet represented an independent transgenic event. Established plants were maintained in M5 media (Khanna et al. 2004) containing hygromycin (10 mg/L) and photographed using a Nikon D3 camera fitted with a Nikon AF-S 24–70 mm f2.8 lens.

### Sequence analysis of CRISPR/Cas9 modified plants

Total DNA was extracted from approximately 20 mg of freshly ground leaf material using the CTAB method (Stewart and Via 1993). Approximately 20 ng of DNA was used as a template for a PCR with 2× 2G Robust HotStart ReadyMix (KAPA) and primers PDS-F2 and PDS-R2. The PCR cycle conditions were as follows: an initial denaturation step at 95 °C for 3 min, 35 cycles of 95 °C for 15 s, 56 °C for 15 s, and 72 °C for 15 s, followed by a final extension at 72 °C for 3 min. The resulting PCR products were gel excised and cloned into pGEM^®^-T Easy (Promega) according to the manufacturer’s specifications. Twelve representative white colonies were selected for each transgenic line. Colonies were inoculated into a PCR containing OneTaq^®^ 2× Master Mix (NEB) and primers M13-F (5′-CCCAGTCACGACGTTGTAAAACG-3′) and M13-R (5′-AGCGGATAACAATTTCACACAGG-3′). PCRs were cycled as described above. A sample (6 µL) of the PCR product was electrophoresed through a 1% TAE agarose gel to confirm the size of the amplicon. PCR products were prepared for sequencing as per NEB protocol with the following modifications. PCR product (2 μL), 1 μL of 5X BrightDye^®^ Sequencing buffer (Molecular Cloning Laboratories), 0.125 μL of Exonuclease I (NEB), 0.25 μL of Shrimp Alkaline Phosphatase (NEB) and 3.625 μL of ddH_2_O were incubated at 37 °C for 45 min followed by inactivation at 80 °C for 15 min. Sequencing reactions were prepared containing 6 μL of enzyme treated PCR product, 1 µL of 5X BrightDye^®^ Sequencing buffer, 0.6 µL of BrightDye^®^ Terminator, 3.2 pmol of M13-F primer and ddH_2_O to a final volume of 20 µL. Thermal cycling was carried out as per BrightDye™Terminator Cycle Sequencing protocol. Products were Sanger sequenced at QUT CARF and analysed using Geneious R11 (http://www.geneious.com, Kearse et al. 2012).

## Results

### Identification of target sequences in the Cavendish PDS gene

**Fig. 1:**
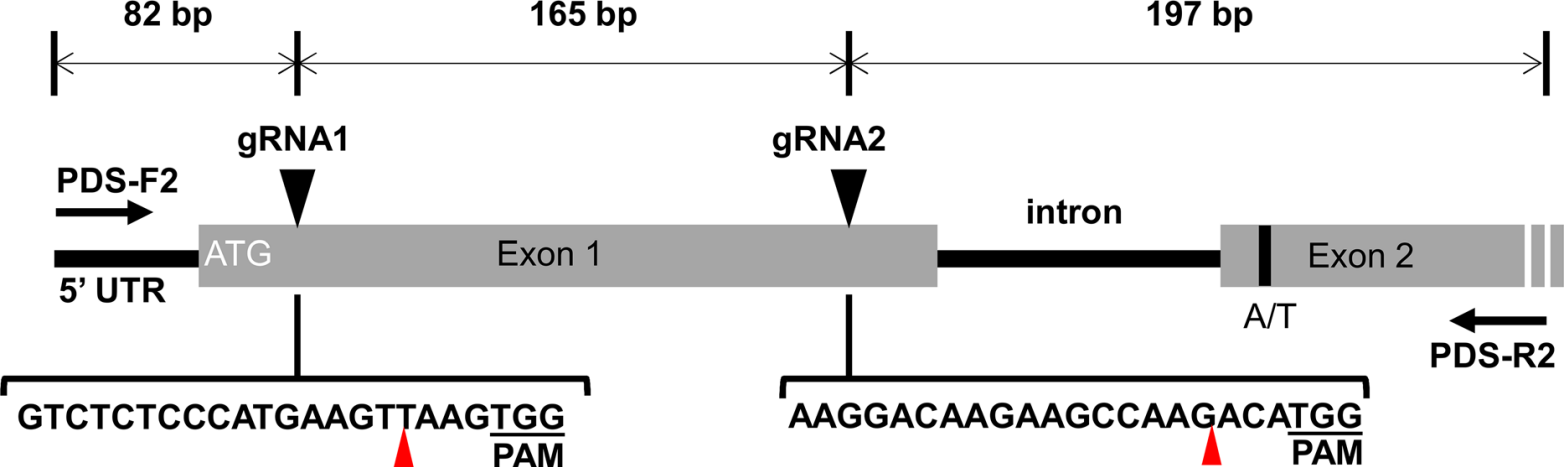
Schematic of the *PDS* gene targeted for editing using CRISPR/Cas9. PDS-F2 and PDS-R2 arrows represent primer binding sites for PCR; gRNA 1 and gRNA 2 represent Target 1 and Target 2 gRNA binding sites, respectively; A/T represents the single nucleotide polymorphism at position + 394 in the PDS alleles; red triangles represent predicted cleavage sites

Using the Cavendish *PDS* cDNA sequence (GenBank Accession JQ762260), primers were designed to amplify a short 5′ segment of the *PDS* gene from cv. Williams leaf gDNA. The resulting product was 444 bp in size and comprised a 47 bp 5′ UTR, 222 bp *PDS* exon 1, 85 bp predicted intron and 90 bp of *PDS* exon 2 (Fig. 1; sequence provided in Appendix 1). Sequencing of 40 pGEM^®^-T Easy clones of the PCR product, showed the 5′UTR and exons were nearly 100% identical to the GenBank accession with only a single nucleotide (A/T) polymorphism at position +394. In total, 60% of clones contained an adenine (A) at this position whereas 40% contained a thymine (T). PDS is likely a single copy gene represented by three single alleles in the Cavendish triploid AAA genome. Therefore, based on sequence percentage we assumed two alleles contained an A and one allele contained a T and are hereafter referred to as 2× A and 1× T, respectively.

We used the WU-CRISPR target prediction server (http://crispr.wustl.edu; Wong et al. 2015) to identify potential target sites in the *PDS* gDNA for CRISPR/Cas9 modification. The two selected target sites differed in their knockout potency scores, Target 1 (a score of 57) and Target 2 (a score of 71), suggesting that the latter would likely be a more effective guide. Simultaneous cleavage of the two target sites by Cas9 was predicted to create a deletion of 165 bp in *PDS* exon 1 (Fig. 1).

### Transformation and regeneration of banana plants with a modified PDS gene

**Fig. 2:**
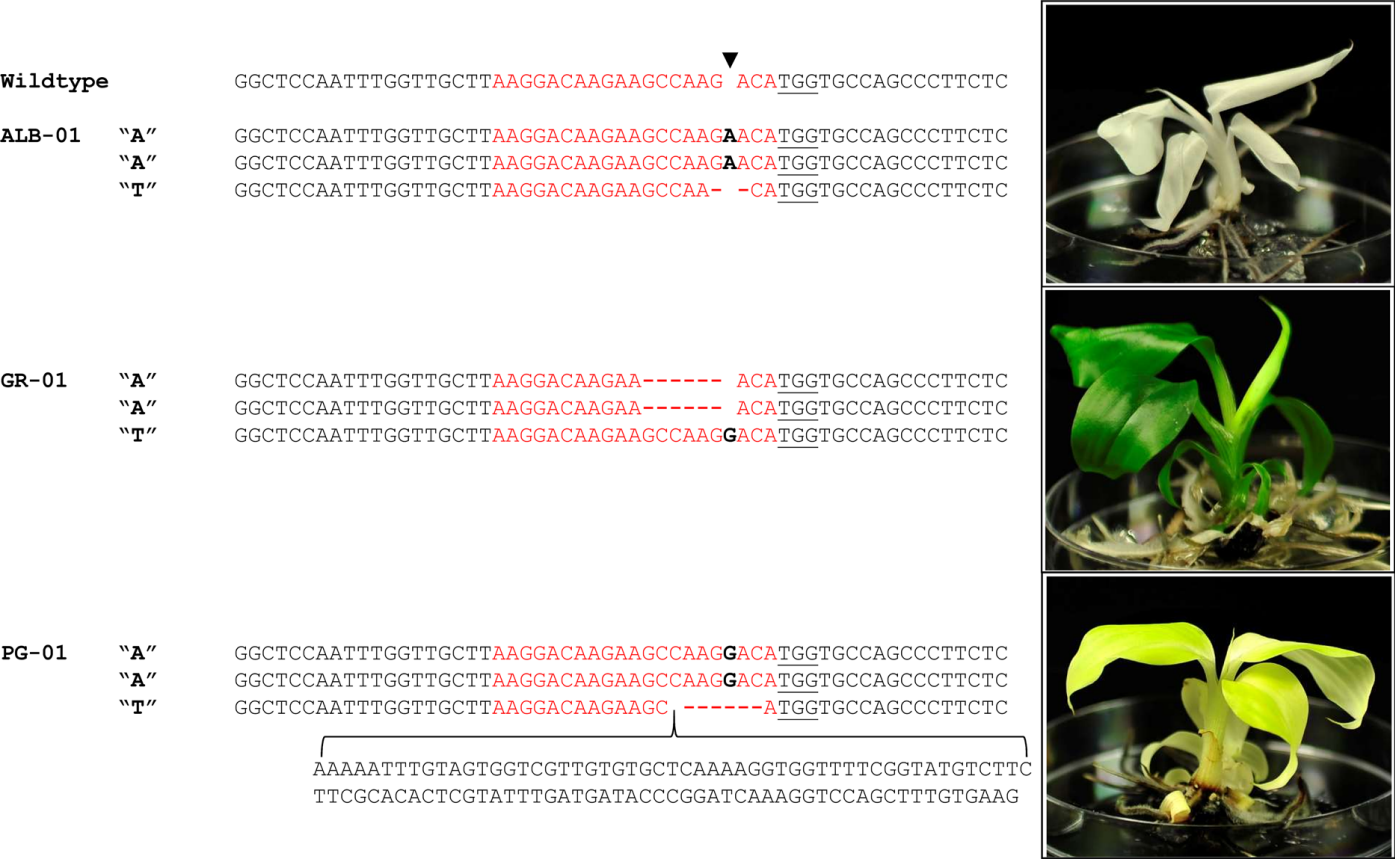
Phenotypes associated with CRISPR/Cas9 editing of the *PDS* gene and representative examples of plant genotype. *ALB* albino, *GR* wild-type green, *PG* pale green; sequence in red indicates the Target 2 gRNA; PAM is underlined; sequence in black bold represent insertions, red dashes represent deletions; black arrow head represents predicted Cas9 cleavage site

*Agrobacterium*-mediated transformation of cv. Williams banana embryogenic cells resulted in the production of 19 transgenic plants, 7 using pUbi-Cas9-PDS and 12 using p35S-Cas9-PDS. The plants were maintained in tissue culture on media supplemented with hygromycin (10 mg/L) and each displayed one of three phenotypes: albino (12/19), dark green (4/19) or pale green (3/19) leaves and pseudostem (Fig. 2, Table 1, Appendix 3), suggesting three different levels of PDS modification. Generally, albino plants developed more slowly than pale or dark green plants, and these events were stunted.

**Table 1:** Summary of PDS modifications at the Target 2 site in banana plants generated using plasmids pUbi-Cas9-PDS or p35S-Cas9-PDS

Phenotype	Line #	Plasmid	Genotype “A” alleles	Genotype “T” allele
Albino Plants	ALB-01	pUbi-Cas9-PDS	+ 1	− 2
	ALB-02	pUbi-Cas9-PDS	− 46	− 13
	ALB-03	pUbi-Cas9-PDS	+ 1	− 13
	ALB-04	p35S-Cas9-PDS	+ 1	− 5
	ALB-05	p35S-Cas9-PDS	+ 1	+ 1
	ALB-06	p35S-Cas9-PDS	− 13	− 2, − 4
	ALB-07	p35S-Cas9-PDS	+ 62	+ 1, + 62
	ALB-08	p35S-Cas9-PDS	− 13, + 1	− 2, + 1
	ALB-09	p35S-Cas9-PDS	− 13, + 1	− 13, + 1
	ALB-10	p35S-Cas9-PDS	− 13, − 6, + 1	+ 1
	ALB-11	pUbi-Cas9-PDS	− 3, + 1, + 6	+ 1
	ALB-12	p35S-Cas9-PDS	− 2, + 1	+ 1, − 13, − 2
Green Plants	GR-01	pUbi-Cas9-PDS	− 6	+ 1
	GR-02	pUbi-Cas9-PDS	+ 6	− 55
	GR-03	p35S-Cas9-PDS	− 6	− 13, − 8
	GR-04	p35S-Cas9-PDS	− 3	− 13, − 1
Pale Green Plants	PG-01	p35S-Cas9-PDS	+ 1	+ 99
	PG-02	p35S-Cas9-PDS	+ 1, + 1	+ 1, + 1
	PG-03	pUbi-Cas9-PDS	+ 1	− 1, + 1

Numbers represent total nucleotide insertions (+) or deletions (−) for independent modifications of either 2× A or 1× T PDS alleles at the Target 2 site

### Analysis of CRISPR/Cas9-mediated editing of the PDS gene

The region spanning the Cas9 target sites was PCR amplified from gDNA isolated from each transgenic plant. The amplicons were cloned into pGEM^®^-T Easy after their apparent sizes had been examined by gel electrophoresis. Simultaneous cleavage of both target sites should produce a 165 bp deletion but none of the amplicons showed the predicted size shift. Nevertheless, sequencing the amplicons revealed the presence of insertions or deletions (indels) ranging from + 105 bp to − 55 bp with 65% of indels observed 0–3 bp upstream of PAM sequence (Table 1). The majority of plants (15/19) were modified only at the Target 2 site and four (ALB-03, ALB-11, GR-02, and PG-03) were modified at both sites, suggesting that CRISPR/Cas9-mediated editing in banana is relatively efficient. Many of the individual plants (6/19) contained more than three distinct PDS modifications. This may reflect ongoing CRISPR/Cas9 modifications in somatic cells, a feature observed in CRISPR/Cas9 modified T_0_ generation rice plants (Zhang et al. 2014), and/or the plants are chimeras of tissues with different indel modifications.

In general, plants displaying a true albino phenotype contained indels in the 2× A and 1× T alleles of the *PDS* gene that each resulted in frameshifting and premature termination. For example, plant ALB-01 contained two distinct modifications of + 1 (2× A alleles) and − 2 bp (1× T allele) at the Target 2 site (Fig. 2; Table 1). While it was not possible to differentiate between the 2× A alleles, it seems likely both contained the same + 1 insertion as no other modifications were detected in the 12 colonies sequenced. The full green plants generally contained an in-frame modification in the 2× A alleles and a disruptive indel in the 1× T allele. For example, plant GR-01 contained an in-frame 6 bp deletion in the 2× A alleles and a 1 bp insertion in the third 1× T allele that caused premature termination of the gene (Fig. 2; Table 1). In contrast, genotyping of plants displaying a pale green phenotype was more difficult to interpret. In the case of PG-01, this plant appeared uniformly pale green and contained a disruptive modification (+ 1 insertion) in the 2× A alleles causing premature termination and an in-frame 99 bp insertion (105 bp insertion and a 6 bp deletion) in 1× T allele (Fig. 2; Table 1). This could result in uniform but weak PDS activity as the modified site is located in the unstructured amino-terminus of the PDS protein, a region likely to accommodate a 33 amino acid insertion without greatly affecting enzyme function (http://www.rcsb.org/). Plants PG-02 and PG-03 were chimeric in both phenotype and genotype, with pale green leaves and pseudostem containing patches of albino cells (Appendix 2) and three or more independent modifications that resulted in disruptive frameshifts to all three alleles (Table 1 and Appendix 3). The predominant modification in these plants was +  frameshifting in all three alleles, which is difficult to correlate with the leaf colour (as it would be expected to produce albinism). Perhaps these plants are initially defective in the 2× A alleles, providing uniform pale green cells, and subsequently lose 1× T allele activity by continued Cas9 editing in random patches of somatic cells.

## Discussion

In order to assess the effectiveness of gene editing in Cavendish banana, we used the CRISPR/Cas9 system to target the endogenous *phytoene desaturase* (*PDS*) gene with a polycistronic gRNA comprising two target sequences in exon 1. This approach generated a 100% *PDS* modification success rate, primarily in the form of small nucleotide indels upstream of the PAM consensus sequence. Indel modifications were detected in multiple allelic copies of the *PDS* gene, and were likely the consequence of the error prone non-homologous end joining repair pathway following CRISPR/Cas9-mediated double stranded DNA breaks; a characteristic observed in a number of other CRISPR/Cas9 modified crops (Fan et al. 2015; Nishitani et al. 2016). No precise dropout deletions were obtained and the frequency of indels differed between target sites and the construct used. The fact that the Target 2 sequence had the highest potency score by WU-CRISPR (http://crispr.wustl.edu) and was edited in every plant suggests that the algorithm is very useful for selecting target sequences in banana. Coupled with improved genomic information, selection of two targets each with WU-CRISPR scores over 70 may be more effective at generating defined deletions. Editing at both target sites only occurred in plants transformed with the pUbi-Cas9-PDS construct. Considering the maize Ubi1 promoter directs expression levels up to 4-fold higher than the CaMV 35S promoter in banana cells (Dugdale et al. 1998), this would suggest that the efficacy of CRISPR/Cas9 editing is proportional to Cas9 abundance. Integration of the CRISPR/Cas9 cassette was confirmed by PCR only; our previous studies have shown a low frequency of transgene integrations (between 1–4 copies) in Cavendish cv. Williams following *Agrobacterium*-mediated (strain AGL1) transformation (results not shown). In a recent transgenic banana field trial by Paul et al. (2017), transgene stability was maintained throughout the life cycle of the plants and over subsequent generations. Likewise, we anticipate CRISPR/Cas9 modifications will be stably inherited in sucker plants propagated vegetatively. Indeed, daughter plants derived from *PDS* modified lines generated in this study displayed the same degree of photo-bleaching as their parent, following in vitro multiplication (Appendix 4).

Notably, 63% of the transgenic plants displayed full albinism from triallelic PDS knockouts and 100% showed some form of Cas9-mediated editing. In a similar study by Kaur et al. (2017), CRISPR/Cas9-mediated editing of *PDS* in the banana cultivar “Rasthali” (Silk AAB group) was significantly less efficient (59% total editing). In this case, a single gRNA targeting PDS exon 5 was used and plants displayed predominantly variegated leaves with few true albino plants surviving tissue culture. Considering the complex polyploid nature of the Cavendish genome and the lengthy time required to generate transgenic banana plants for field analysis, a highly efficient editing platform for creating triallelic homozygous modifications is essential for the future improvement of this cultivar.

The uniform monoculture of Cavendish makes it particularly susceptible to diseases for which it has no natural resistance. Bananas are prone to a number of important disease threats already that can significantly reduce fruit yields and decimate crops, including bunchy top disease caused by *banana bunchy top virus*, Black and Yellow Sigatoka caused by *Mycosphaerella* spp., Bacterial wilt caused by *Xanthamonas campestris* pv. *musacearum* and the devastating Panama disease or Tropical Race 4 caused by *Fusarium oxysporum* f. sp. *cubense*. With ongoing gene discovery in a range of related sub-species there is potential to engineer Cavendish resistant to multiple diseases. The establishment of an efficient gene editing platform that can manipulate endogenous disease resistance genes and up- or down- regulate their expression patterns, by either knock-out or knock-in modifications, is a significant step towards achieving these goals.

## Figures and Tables

**Fig. 3:**
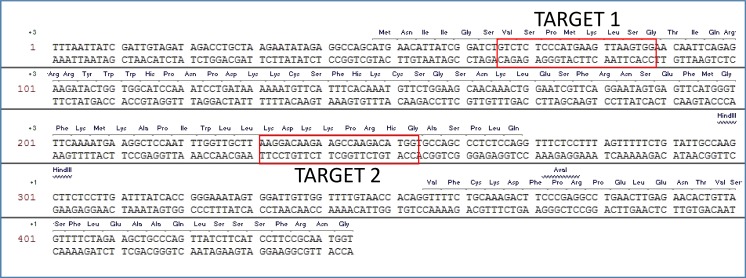
Sequence of the Cavendish cv. “Williams” *PDS* gene fragment targeted for editing

**Fig. 4:**
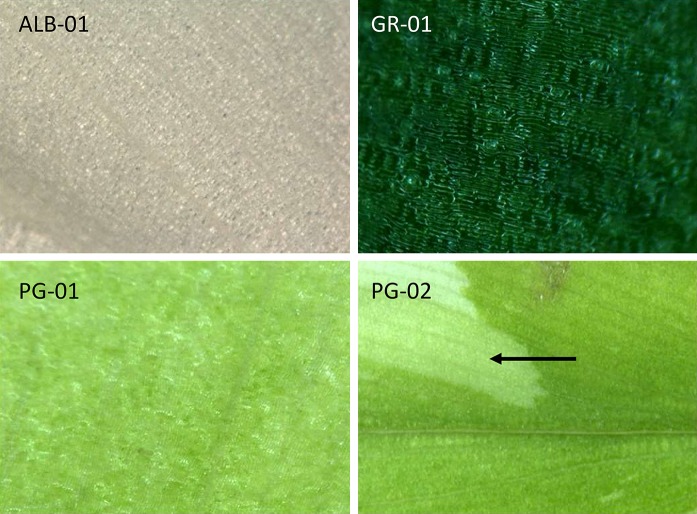
Leaf colour phenotypes associated with CRISPR/Cas9 modification of the *PDS* gene. *ALB* albino, *GR* wild-type green, *PG* pale green. Black arrow designates albino patch of cells on pale green leaf background

**Fig. 5:**
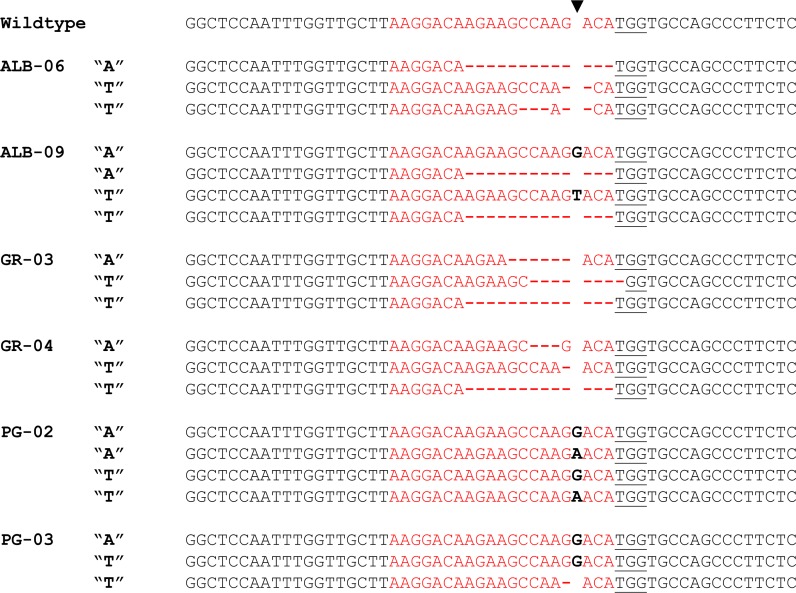
Sanger sequence trace of representative CRISPR/Cas9 edited albino, pale green and wild-type green plants

**Fig. 6:**
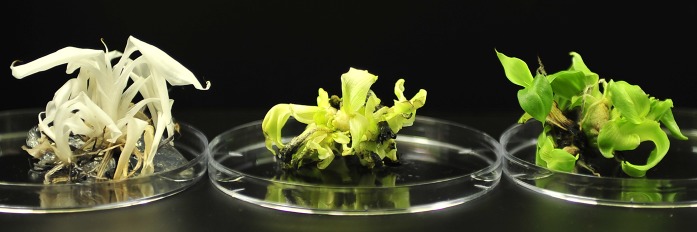
Stable inheritance of PDS modifications causing albino, pale green and wild-type green phenotypes in daughter plants following in vitro multiplication

## Appendix 1

See Fig. 3.

## Appendix 2

See Fig. 4.

## Appendix 3

See Fig. 5.

## Appendix 4

See Fig. 6.
